# Assessment of the Quality and Readability of Web-Based Arabic Health Information on Halitosis: Infodemiological Study

**DOI:** 10.2196/54072

**Published:** 2024-08-28

**Authors:** Khalid Aboalshamat

**Affiliations:** 1 Preventative Dentistry Department College of Dental Medicine Umm Al-Qura University Makkah Saudi Arabia

**Keywords:** halitosis, bad breath, malodor, Arabic web-based, infodemiological study, oral malodor, readability, infodemiology, health information, Arabic mouth medical information, reliable information, odor treatment

## Abstract

**Background:**

Halitosis, characterized by an undesirable mouth odor, represents a common concern.

**Objective:**

This study aims to assess the quality and readability of web-based Arabic health information on halitosis as the internet is becoming a prominent global source of medical information.

**Methods:**

A total of 300 Arabic websites were retrieved from Google using 3 commonly used phrases for halitosis in Arabic. The quality of the websites was assessed using benchmark criteria established by the *Journal of the American Medical Association*, the DISCERN tool, and the presence of the Health on the Net Foundation Code of Conduct (HONcode). The assessment of readability (Flesch Reading Ease [FRE], Simple Measure of Gobbledygook, and Flesch-Kincaid Grade Level [FKGL]) was conducted using web-based readability indexes.

**Results:**

A total of 127 websites were examined. Regarding quality assessment, 87.4% (n=111) of websites failed to fulfill any *Journal of the American Medical Association* requirements, highlighting a lack of authorship (authors’ contributions), attribution (references), disclosure (sponsorship), and currency (publication date). The DISCERN tool had a mean score of 34.55 (SD 7.46), with the majority (n=72, 56.6%) rated as moderate quality, 43.3% (n=55) as having a low score, and none receiving a high DISCERN score, indicating a general inadequacy in providing quality health information to make decisions and treatment choices. No website had HONcode certification, emphasizing the concern over the credibility and trustworthiness of these resources. Regarding readability assessment, Arabic halitosis websites had high readability scores, with 90.5% (n=115) receiving an FRE score ≥80, 98.4% (n=125) receiving a Simple Measure of Gobbledygook score <7, and 67.7% (n=86) receiving an FKGL score <7. There were significant correlations between the DISCERN scores and the quantity of words (*P*<.001) and sentences (*P*<.001) on the websites. Additionally, there was a significant relationship (*P*<.001) between the number of sentences and FKGL and FRE scores.

**Conclusions:**

While readability was found to be very good, indicating that the information is accessible to the public, the quality of Arabic halitosis websites was poor, reflecting a significant gap in providing reliable and comprehensive health information. This highlights the need for improving the availability of high-quality materials to ensure Arabic-speaking populations have access to reliable information about halitosis and its treatment options, tying quality and availability together as critical for effective health communication.

## Introduction

Halitosis, bad breath or oral malodor, is a condition characterized by an undesirable mouth odor [1,2]. The primary factors contributing to 80%-90% of halitosis cases are intraoral in nature, which include inadequate oral hygiene practices, a coated tongue, and periodontal conditions [3]. Halitosis is a common problem worldwide, with a prevalence of 66% in China [4], 53.5% in northern Italy [5], and 32.5% in Switzerland [6]. Also, studies in Arab countries found a prevalence ranging from 6.2% to 68.5% in Saudi Arabia [7-9] and 78% in Jordan [10]. Halitosis is called a “social life killer” due to its effect on relationship breakups, social awkwardness, rejection of job and career opportunities, and even business problems [11]. Halitosis has been found to lower the quality of life, affecting marriage, intimate relationships, friendships, and daily social interactions [11]. These consequences can severely impact an individual’s social life. Beyond the immediate social impact, persistent halitosis can signal underlying health issues, including oral infections and systemic conditions such as Parkinson disease, allergic rhinitis, *Helicobacter pylori* gastric infection, viral hepatitis B [3], and even COVID-19 [9].

There are different professional methods to deal with halitosis such as dental treatment, mouthwash, and toothpaste to eliminate the source of bacterial problems [12]. Probiotics have been found effective in managing halitosis despite the need for further clinical evidence [13] underscoring the importance of continuing research and clinical trials to validate their efficacy. Similarly, tongue scraping has been found to provide only short-term reduction in volatile sulfur compounds and halitosis [14]. Nevertheless, many patients tend to address halitosis with home remedies such as honey and anise [9], and other herbal remedies that are popular due to their low cost and minimal side effects [12]. People usually use the internet as a source of information to find home remedies [15].

In recent times, accessing medical information through the internet has become a prominent global trend [16-18]. Notably, a substantial percentage of patients (ranging from 45% to 85%) bring information sourced from their web-based searches when they consult with health care providers [19]. This inclination toward web-based health information-seeking has been further amplified by the recent COVID-19 pandemic, compelling individuals to turn to the internet to address their queries [20-22]. However, a critical issue that looms large is the proliferation of misinformation on the internet, a concern documented as a worldwide phenomenon [23,24]. This poses a significant threat to individuals’ quality of life and even mortality [23], necessitating the implementation of stringent regulations, heightened public awareness, and enhanced dissemination of reliable health-related information [24].

As a result, numerous papers evaluating the quality and readability of web-based information on oral health conditions and diseases have been published in English. Examples of these papers include studies on halitosis [25], medication-related osteonecrosis of the jaw [26], postendodontic treatment selection [27], and treatment of the mouth in systemic sclerosis [28]. Analogous research was carried out in other languages, including Danish [29] and Spanish [30], suggesting the significance of additional languages. However, there is limited research available on oral health topics in Arabic, assessing general content to the public. A few studies have investigated oral cancer [31], dental implants [32], periodontal disease [33], and denture hygiene information [34]. The majority of these studies used comparable approaches, including the readability calculator tool, the DISCERN tool [35] to assess website quality, and the existence of the Health on the Net Foundation Code of Conduct (HONcode) [36]. Even though halitosis is considered the third most common reason for seeking dental treatment [11], no research has been done to evaluate the quality and readability of halitosis information in Arabic. In the literature, there is a single study that assesses the quality and readability of halitosis but only in English [25], which highlights the gap in knowledge. Additionally, there are 22 nations with Arabic as their official language, and more than 422 million people speak it [37]. Thus, this study aims to assess the quality and readability of web-based Arabic health information on halitosis. The alternative hypothesis for this study is that there is a significant association between the quality and readability of web-based Arabic health information on halitosis.

## Methods

### Search Strategy

This research falls under the domain of infodemiological investigation (study of web-based information’s impact on health). The study was conducted using Google Chrome, specifically version 114.0.5735.6. The search was done in incognito mode. This approach was used to mitigate the impact of search history and tailor the search algorithms to customize the search results [38]. The search was carried out using widely used search engines such as Google. The search was conducted on May 7, 2023. Google was used exclusively because it is the world’s leading search engine.

**Figure 1:**
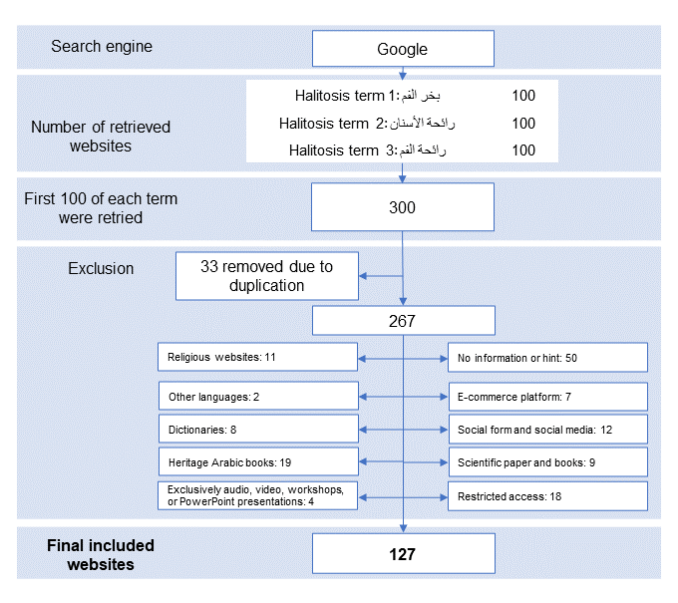
Flowchart illustrating the hierarchical structure and interconnectedness of the websites discovered through the search method used.

Three synonymous terms for halitosis, both in formal and informal Arabic, were used, which translate to “halitosis,” “mouth breath,” and “teeth smell” in English. These terms in Arabic are “رائحة الأسنان,” “**رائحة الفم**,” and “**بخر الفم**.” A total of 300 web pages were obtained by retrieving the first 100 websites for each phrase. All instances of duplicated websites were removed. The previous points outline the inclusion criteria for the website; however, the selection of websites for this research was determined based on the following exclusion criteria: sources in languages other than Arabic; dictionaries; Arabic books published before the 18th century; proprietary commercial merchandise available for purchase on e-commerce platforms such as Amazon or Noon platforms; social forums and social media websites; academic scientific papers or textbooks; websites that are prohibited or have restricted access, necessitating the use of identification and a password; sources with no information about halitosis or information presented only in hints; religious websites that ask for religious clarification “Fatwa” regarding halitosis; and content delivered exclusively through video, audio, or PowerPoint presentations. The researchers applied these criteria to ensure that only relevant and trustworthy material was included in the study. This selection process is delineated in Figure 1.

Each website was categorized in terms of specialization, affiliation, and content type [39]. Specialization varied from being partial or exclusive to the topic. The affiliation had 5 different classes: nonprofit organization, university or medical center, government, commercial, and journalism. In terms of content types, the website could provide clinical trials, medical facts, human stories, or questions and answers. Additionally, each website was noted to include video, audio, and images on each website was documented.

### Quality Assessment

To evaluate website quality, a comprehensive evaluation was conducted by 2 assessors, denoted as RA and AA, both of whom are qualified professionals as dentists. They used the DISCERN [35] and *Journal of the American Medical Association* (*JAMA*) tools [40] in this assessment. The calibration process consisted of 2 parts. In the first stage, each assessor independently evaluated 10 websites. Any discrepancies in their assessments were subsequently addressed through discussions with the lead investigator. A further evaluation of 20 websites was carried out, and any disagreements were resolved through collaborative efforts with a discussion with the lead investigator. This was to ensure calibration. For the following website, any disagreements were verified by the lead investigator as a final decision. The DISCERN instrument comprises a set of 16 questions categorized into 3 main sections. The first section, questions 1-8, aims to assess the credibility of websites as sources of information for various medical topics. The next section, including questions 9-15, focuses on various aspects of therapeutic options. Question 16 serves as an overall evaluation criterion for quality. The theoretical score range of the DISCERN instrument spans from 16 to 80. Websites scoring between 16 and 32 are classified as having poor quality, those scoring between 33 and 64 are considered to have moderate quality, and websites that score 65 or greater are classified as high quality.

The assessment of each website was conducted using the *JAMA* criteria for website evaluation [40], the presence of the HONcode [36], and the DISCERN tool [35]. The *JAMA* benchmarks encompass four primary criteria that must be met: (1) authorship, which involves the inclusion of authors’ contributions, affiliations, and their relevant credentials; (2) attribution, which entails providing references and citations; (3) disclosure, which requires indicating any sponsorship, ownership, commercial funding, advertising, or potential conflicts of interest; and (4) currency, which necessitates the inclusion of the publication date and information on updates.

Each website received a score of 1 point if it met one of the criteria; otherwise, it received a score of 0. The *JAMA* benchmark score was calculated as the cumulative total of the preceding categories, which ranged from 0 to 4 points, with 4 being the maximum score attainable. The HONcode tool allows websites to obtain authorization to display a stamp, similar to a HON award badge, on their web page, contingent upon their adherence to the HONcode standards, which is valid for a period of 1 year.

### Readability Assessment

The notion of readability refers to the systematic use of formulas to determine the level of reading comprehension required for an individual to understand written materials [41]. The readability of the content was evaluated using a freely available readability calculator [42], which is commonly used to measure the readability of English text. This calculator was the standard method of measurement by literature for similar studies to assess dental English websites [25,27,28] and Arabic dental websites [31-34]. This calculator uses 3 indices: the Flesch-Kincaid Grade Level (FKGL), the Simple Measure of Gobbledygook (SMOG), and the Flesch Reading Ease (FRE). The FKGL uses metrics such as average sentence length and average syllables per word to determine the level of reading complexity. In contrast, the SMOG Index calculates the ratio of words that have 3 or more syllables. As the score increases, the reading difficulty also increases. According to the previous literature [43,44], the FKGL and SMOG readability scores should ideally be around 7. The FRE algorithm computes a numerical score between 0 and 100 by analyzing the mean sentence length and the mean syllable count per word. A score of 80 or greater on the FRE scale suggests content that is easily comprehensible [43,44].

### Statistical Analysis

The process of data entry and cleansing was conducted using the Microsoft Excel program. The data analysis was carried out using SPSS version 29 (IBM Corp). Descriptive statistics were used to analyze the data collected from websites, using measures such as count, percentages, mean, SD, median, minimum, maximum, and IQR. The Spearman correlation coefficient was used to evaluate the association between *JAMA*, DISCERN scores, and readability indices. This is because all the correlation tests conducted violated the normality assumption of the dependent variables (number of words, number of sentences, FKGL, SMOG, and FRE). The *P* values of the Shapiro-Wilk tests for the aforementioned variables were *P*<.001. A predetermined *P*=.05 was used as the threshold for determining statistical significance. Raw data are available in Multimedia Appendix 1.

**Ethical Considerations**

Given that this is an infodemiological study and does not involve data collection from humans or animals, no institutional review board approval or ethical application was required. Consequently, no submission was made to the ethics committee at Umm Al-Qura University, Saudi Arabia.

## Results

### Arabic Halitosis Websites

Of the 300 websites about halitosis, only 267 websites remained after removing the duplication (n=33, 11%), as seen in Figure 1. The excluded websites encompassed a diverse range of categories, including those in other languages (n=2, 0.6%), dictionaries (n=8, 2.6%), heritage Arabic books (n=19, 6.3%), e-commerce platforms (n=7, 2.3%), social forums and social media websites (n=12, 4%), academic scientific papers or textbooks focused on halitosis (n=9, 3%), websites with restricted access (n=18, 6%), as well as those offering either no information or mere hints (n=50, 16.6%). Additionally, a small number solely used video, audio, or Microsoft PowerPoint presentations (n=4, 1.3%), and some websites focused on religious inquiry “Fatwa” regarding halitosis (n=11, 3.6%). The classification of the Arabic halitosis websites in terms of content type, affiliation, specialization, and inclusion of image, video, and audio content is displayed in Table 1. Most of the Arabic websites on halitosis predominantly feature medical facts, with 126 (99.2%) sites focusing exclusively on this content type. Almost all sites (n=125, 98.4%) are exclusively related to halitosis. In terms of affiliation, journalism and commercial entities are the leading sources, comprising 41.7% (n=53) and 37.8% (n=48) of the websites, respectively. The inclusion of multimedia content is notable, with 81.1% (n=103) of the sites containing images, but videos (n=10, 7.8%) and audio (n=4, 3.15%) are less commonly featured.

**Table 1:** Classification of the Arabic halitosis websites in terms of content type, affiliation, specialization, and inclusion of image, video, and audio content.

Item		Websites, n (%)
**Content type**		
	Medical facts	126 (99.2)
	Human interest stories	1 (0.79)
	Question and answers	0 (0)
	Clinical trials	0 (0)
**Specialization**		
	Exclusively related to halitosis	125 (98.4)
	Partly related to halitosis	2 (1.5)
**Affiliation**		
	Journalism	53 (41.7)
	Commercial	48 (37.8)
	University or medical center	19 (14.9)
	Government	4 (3.1)
	Nonprofit organization	3 (2.3)
**Contain image**		
	Yes	103 (81.1)
**Contain video**		
	Yes	10 (7.8)
**Contain audio**		
	Yes	4 (3.1)

### Assessment of the Quality of Arabic Halitosis Websites

When evaluating the *JAMA* benchmark criteria, it was found that only 2.3% (n=3) met the requirements for authorship, 3.1% met the criteria for attribution, 0.7% (n=1) met the standards for disclosure, and 9.4% (n=12) met the criteria for currency, as indicated in Table 2. The majority of websites (n=111, 87.4%) did not meet any of the criteria, 9.4% (n=12) met 1 criterion, and 3.1% (n=4) met 2 criteria. No website reached a score of 3 or the maximum of 4 based on the *JAMA* benchmark criteria. The mean for the *JAMA* benchmark criteria was 0.15 (SD 0.44), and the median was 0 (IQR 0-0). All the websites lacked HONcode certification.

According to the DISCERN score classification, none of the websites achieved a high-quality score (ie, 65 or greater). A total of 72 (56.6%) websites received a moderate quality score (between 33 and 64), while 55 (43.3%) obtained a low-quality score (between 16 and 32), as indicated by the scores for each item shown in Table 3. The mean for the total DISCERN score was 34.55 (SD 7.46). The criteria best addressed were relevance (Q3: 4.91, SD 0.43) and clarity of treatment choices (Q14: 4.01, SD 1.55), whereas the least addressed were treatment impact on quality of life (Q13: 1.02, SD 0.18) and addressing uncertainties (Q8: 1.03, SD 0.35).

**Table 2:** Assessment of the quality of Arabic halitosis websites using JAMA^a^ benchmark criteria.

*JAMA* criteria	Website, n (%)
Authorship	3 (2.3)
Attribution	4 (3.1)
Disclosure	1 (0.7)
Currency	12 (9.4)
0 *JAMA* criteria	111 (87.4)
1 *JAMA* criterion	12 (9.4)
2 *JAMA* criteria	4 (3.1)
3 or 4 *JAMA* criteria	0 (0)

^a^JAMA: Journal of the American Medical Association.

**Table 3:** DISCERN assessment of Arabic halitosis websites.

Domain and question		Score, mean (SD)
**Reliability**		
	Q1. Are the aims clear?	1.43 (0.86)
	Q2. Does it achieve its aims?	1.72 (1.48)
	Q3. Is it relevant?	4.91 (0.43)
	Q4. Is it clear what sources of information were used to compile the publication?	1.83 (1.17)
	Q5. Is it clear when the information used or reported in the publication was produced?	2.51 (1)
	Q6. Is it balanced and unbiased?	3.68 (0.95)
	Q7. Does it provide details of additional sources of support and information?	2.29 (1.7)
	Q8. Does it refer to areas of uncertainty?	1.03 (0.35)
**Treatment options**		
	Q9. Does it describe how each treatment works?	3.00 (1.51)
	Q10. Does it describe the benefits of each treatment?	2.06 (1.15)
	Q11. Does it describe the risks of each treatment?	1.28 (0.7)
	Q12. Does it describe what would happen if no treatment is used?	1.16 (0.69)
	Q13. Does it describe how the treatment choices affect the overall quality of life?	1.02 (0.18)
	Q14. Is it clear that there may be more than 1 possible treatment choice?	4.01 (1.55)
	Q15. Does it provide support for shared decision-making?	1.35 (0.95)
	Q16. Overall quality of the publication as a source of information about Halitosis.	1.28 (0.83)

### Readability of Arabic Halitosis Websites

Using the readability calculator, the median, IQR, minimum, and maximum were recorded for different items as shown in Table 4. The readability assessment showed a median of 3164 (IQR 2024-4618) characters (without spaces) and 669 (IQR 412-1025) words per paper, with content typically comprising 30 sentences. The readability assessments revealed that the majority of the materials (n=86, 67.7%) have an FKGL below 7, with 41 materials (32.2%) scoring 7 or more, indicating easier comprehension. Meanwhile, the SMOG and FRE scores suggest a high readability level, with 98.4% (n=125) of materials categorized below a SMOG score of 7 and 90.5% (n=115) having an FRE score of 80 or higher. There were only 1.5% (n=2) of the material categorized as equal or greater SMOG score of 7, and 9.4% (n=12) having an FRE score below 80.

Spearman correlation coefficients were computed to assess the relationships between the DISCERN score and various readability indices, including the number of words, number of sentences, FKGL, SMOG, and FRE, as presented in Table 5.

**Table 4:** Readability of Arabic halitosis websites.

Item	Score	
	Median (IQR)	Minimum-maximum
Number of characters (without spaces)	3164 (2024-4618)	416-32,898
Number of words	669 (412-1025)	86-6929
Number of sentences	30 (15-40)	2-325
Average number of characters per word	4.75 (4.6-4.8)	4.08-5.19
Average number of syllables per word	1 (1-1.01)	1-1.1
Average number of words per sentence	22.91 (19.1-29.6)	12.12-611.5
FKGL^a^	5.16 (3.8-7.7)	0.96-234.71
SMOG^b^	3.71 (3-4.3)	3-7.74
FRE^c^	98.64 (92-101.9)	8.05-498.58

^a^FKGL: Flesch-Kincaid Grade Level.

^b^SMOG: Simple Measure of Gobbledygook.

^c^FRE: Flesch Reading Ease.

**Table 5:** Assessing the relationship between halitosis websites’ DISCERN score and readability indices using Spearman correlation.

		DISCERN	Number of words	Number of sentences	FKGL^a^	SMOG^b^	FRE^c^
**DISCERN**							
	Spearman ρ	1	0.433	0.433	–0.123	0.176	0.092
	*P* value	—^d^	<.001	<.001	.17	.047	.30
**Number of words**							
	Spearman ρ	0.433	1	0.773	0.043	0.146	0.048
	*P* value	<.001	—	<.001	.63	.10	.59
**Number of sentences**							
	Spearman ρ	0.433	0.773	1	–0.482	0.063	0.402
	*P* value	<.001	<.001	—	<.001	.49	<.001
**FKGL**							
	Spearman ρ	–0.123	0.043	–0.482	1	0.151	–0.895
	*P* value	.17	.63	<.001	—	.09	<.001
**SMOG**							
	Spearman ρ	0.176	0.146	0.063	0.151	1	–0.208
	*P* value	.047	.10	.49	.09	—	.02
**FRE**							
	Spearman ρ	0.092	0.048	0.402	–0.895	–0.208	1
	*P* value	.30	.59	<.001	<.001	.02	—

^a^FKGL: Flesch-Kincaid Grade Level.

^b^SMOG: Simple Measure of Gobbledygook.

^c^FRE: Flesch Reading Ease.

^d^Not applicable.

## Discussion

### Overview

While halitosis is one of the most common problems in dental care [11], the internet is considered to be a main source of information to the public [45] despite the quality and overwhelming quantity of information. This study aimed to assess the quality and readability of web-based Arabic health information on halitosis. Despite the high number of sites regarding halitosis in Arabic, our results revealed that the Arabic halitosis websites are highly readable but poor in quality from a professional and academic point of view.

### Quality Assessment of Arabic Halitosis Websites

DISCERN score was approximately similar to a previous study about halitosis [25] and other previous Arabic infodemiological studies that investigated denture hygiene [34], dental implants [32], and periodontal diseases [33]. There were small noncrucial differences, and the scores ranged between the low and moderate categories of DISCERN. However, our DISCERN result was lower than the other infodemiological studies that assessed the quality and readability of third molar tooth websites in English [46]. In this study, not even a single website scored high according to the DISCERN cutoff. On the other hand, the *JAMA* score in this study was found to be lower than all the previous studies about English halitosis websites [25] or other Arabic dental infodemiological studies [32-34]. It should be noted that the previous study about English halitosis websites fulfilled the criteria of authorship, attribution, disclosure, and currency by 51.8%, 35.6%, 29%, and 48.5%, which is far higher than the percentages of such items in our studies. This leads to the conclusion that the quality of available material on Arabic websites on dental topics is generally not satisfactory, and the quality of websites about halitosis is much poorer in Arabic language than in English although both are unsatisfactory. This absence of high-quality Arabic websites shows the need for the development of comprehensive and reliable web-based dental resources. It is possible to use DISCERN items as guidelines to develop better Arabic content about halitosis. This is especially true for the items that scored low in the DISCERN assessment, which deals with uncertainty and on how treatment affects the quality of life. The English websites with higher scores in dental topics are not an odd finding. This is because DISCERN is available mainly for English websites. However, collaborative initiatives involving English content creators and dental associations in Arabic-speaking regions can be established to address these gaps.

There was no single website in this study that had the HONcode certification, which is somewhat similar to other infodemiological studies about dental websites in Arabic, which reported less than 2 websites having HONcode [32-34]. On the other hand, 14.9% of websites about halitosis in English had HONcode [25], with similar percentages in other infodemiological studies about dental issues in English [47-49]. One of the explanations to this is that HONcode might be familiar among websites in English, and that granting the certification might be more convenient to English websites rather than those in Arabic. In fact, the HONcode instruction is given in English only. It is recommended that HONcode certification be introduced to medical and dental website content creators or that a similar certificate be made available that is assessed in Arabic.

### Readability Assessment of Arabic Halitosis Websites

With regard to readability, this study indicated that the majority of websites were readable for those possessing language skills beyond the sixth grade level. This aligned with other studies that assessed previous Arabic dental websites [32-34]. This might not be the case in the previous study of halitosis in English [25], and other dental topics in English also [28] found that the level of reading is more challenging. These results did not find a relation between FKGL, SMOG, and FRE with DISCERN scores. This refutes a proposed explanation that the websites were created simply and the quality of the websites was reduced. However, there are 2 possible explanations for this. First, the Arabic content creator wrote in simple language to reach a public audience compared to the English websites. Second, the language calculator was designed for the English language in the United States and might not be very accurate when used for other languages, despite being used by previous studies [32-34]. Such aspects need to be verified by future studies and include professional assessment using the common method to assess the readability of Arabic content.

### Contents of Arabic Halitosis Websites

As the previous aspects investigated Arabic halitosis websites’ contents and readability, the assessment did not include the verification of the contents’ validity as done in other studies [50,51]. Merely observing the presence or absence of DISCERN quality items might not sufficiently judge the validity and robustness of the information provided. Referencing evidence-based resources about halitosis [52,53] can offer a better assessment of new information related to halitosis. Furthermore, leveraging artificial intelligence could be a promising future direction as a tool to provide deeper insights into the content of such websites [54]. Artificial intelligence and natural language processing technologies can aggregate and process content from specified websites, enabling researchers to categorize the information into accurate, uncertain, and incorrect categories. Following this classification, a detailed analysis of the website content can be conducted. However, implementing such a methodology necessitates a comprehensive and nuanced strategy.

One of the notable aspects of this study is that there are a considerable number of websites (n=19, 6.3%) displaying heritage Arabic books discussing halitosis that were published centuries ago. For example, there was a book that was published in 776 Hijri, that is, AD 1398, and another book that was published in AD 1505. Also, there were 11 websites that had religious clarifications (Fatwa) regarding halitosis. This can be understood, as Islamic teachings have some specific teachings regarding halitosis; for instance, Muslims are encouraged to use *Salvadora persica* (siwak) for good oral breath [55]. Also, people who have halitosis are discouraged from praying in a mosque. In fact, halitosis is a serious issue not only in Islam; for example, according to the Talmud, a holy book for Jews, halitosis can also be a cause of divorce [56]. Such aspects might be important as a social-cultural dimension when crafting website content suitable for the audience.

In this study, social media results were excluded from the investigated websites. Social media often uses short text and might depend on continuous dialogue that does not belong to a single entity, which makes it very difficult to assess using the existing tools. However, further assessments can be conducted on social media using appropriate assessment tools.

This study might be one of the few studies that assessed the quality and readability of dental problems in Arabic, using a well-known validated tool. One of the limitations of this study is that the validity and the contents of the Arabic halitosis websites were not assessed in comparison to scientific evidence-based resources. It is not known according to the assessment if the current website provides sound or misleading information. The importance of such an aspect is accentuated in this era of massive health misinformation [23,24]. Future studies should be directed to conduct content analysis and verify the trustworthiness of the information on these websites. Also, there are studies that introduce different approaches to deal with halitosis [3,13] such as probiotics and others that should be included in the websites for the public in order to provide thorough content. Also, the website search was conducted over a desktop computer, which might be different if we conducted the search over a smartphone [57], especially in this new era where people search for information on mobile devices from anywhere and at any time.

### Study Limitations

The websites retrieved in this study were taken from 1 search engine, unlike some of the previous studies. The reason might be because Google has been the main search engine for the majority of people worldwide over the last 10 years, with a large difference compared to other search engines [58]. However, this limitation should be considered in future research, and other search engines should be investigated, such as Bing and Yahoo. Finally, this study treated Arabic websites as a homogeneous group, not accounting for the geographical diversity among Arabic speakers that could influence digital health information. This diversity might result in digital content variations related to culture, region, and accessibility. Consequently, this could affect the generalizability of our findings.

### Conclusions

While the readability of halitosis and other dental topic websites in Arabic was very good, their quality was poor. It is essential to make reliable information accessible for understanding halitosis and other dental topics, as well as treatment options, for Arabic-speaking populations. Collaborative efforts should be directed toward formulating such content, especially considering recent advancements in treatment modalities. Future studies should investigate the content of dental websites in Arabic to assess the validity of information using evidence-based approaches.

## Appendix

Multimedia Appendix 1 Raw data.

## Abbreviations

FKGL: Flesch-Kincaid Grade Level
FRE: Flesch Reading Ease
HONcode: Health on the Net Foundation Code of Conduct
JAMA: Journal of the American Medical Association
SMOG: Simple Measure of Gobbledygook
